# Prevalence and Risk Factors for Acetabular Rim Irregularities in Infants with Hip Dysplasia: A Cohort Study

**DOI:** 10.3390/jcm15166136

**Published:** 2026-08-07

**Authors:** Matthias Pallamar, Philipp Scheider, Carina Weiß, Florentin Spalt, Gudrun H. Borchert, Catharina Chiari

**Affiliations:** 1 Department of Pediatric Orthopaedics and Foot Surgery, Orthopaedic Hospital Speising, Speisinger Straße 109, 1130 Vienna, Austria; philipp.scheider@oss.at (P.S.); carina.weiss@oss.at (C.W.); catharina.chiari@oss.at (C.C.); 2 Medical Campus, Medical University of Vienna, Spitalgasse 23, 1090 Vienna, Austria; 3 Herz-Jesu-Hospital, Baumgasse 20A, 1030 Vienna, Austria; florentin.spalt@kh-herzjesu.at; 4 Dr. Borchert Medical Information Management, Egelsbacher Str. 39e, 63225 Langen, Germany; gudrun.borchert@borchert-medical.com

**Keywords:** hip ultrasound, infants, sonographic acetabulum rim irregularities, SARI, developmental dysplasia of the hip, DDH, prevalence, associated factors

## Abstract

**Background/Objectives**: Hip ultrasound is the standard screening method for developmental dysplasia of the hip (DDH) in infants, yet sonographic acetabular rim irregularities (SARIs) remain understudied regarding their prevalence and clinical associations. **Methods**: A total of 1010 infants within the first four months of life, referred to a specialized pediatric orthopedic hip clinic between 2014 and 2019 for suspected or confirmed DDH, were included in this retrospective cohort study. Hip ultrasound was performed using the standardized Graf technique. Clinical, demographic, and sonographic data were extracted from patient records. Two pediatric orthopedic specialists assessed all ultrasound hip images for the presence of an SARI and measured its relative size (d/D). Associations among SARI, patient characteristics, and sonographic parameters were analyzed using appropriate univariate and multivariable statistical methods (*p* < 0.05). **Results**: We identified SARI in 9.11% of all hips and in 13.07% of all infants, with many developing during Pavlik harness treatment. Univariate and multivariate analyses revealed cesarean delivery as an independent factor associated with SARI occurrence. SARIs that developed over time had a significantly larger d/D ratio compared with those that were visible from the very beginning (*p* = 0.04). **Conclusions**: These findings establish a baseline prevalence and highlight potential risk markers for SARI in a dysplasia-enriched cohort. Further prospective studies are warranted to elucidate the clinical significance of SARI and its impact on treatment outcomes and hip development.

## 1. Introduction

Hip ultrasound is a well-established diagnostic procedure used in screening for developmental dysplasia of the hip (DDH) in infants. This examination describes the position of the femoral head relative to the acetabulum to detect changes in the acetabulum, possibly in conjunction with lateralization or dislocation of the femoral head. The shape of the bony acetabular roof can also be assessed using ultrasound. Of particular interest here is the shape of the lateral margin of the acetabulum—the “acetabular rim”. This area plays a crucial role in assessing the morphology of the roof of the acetabulum and measuring the proportion of the femoral head covered by it. Together, the morphology and measurement of the acetabular roof influence the treatment planning for DDH in infants. Various hip ultrasound examination methods essentially aim to describe the boundary between the bony and cartilaginous portions of the acetabulum regarding angular degrees or percentages [1,2,3,4,5]. Therefore, it cannot be ruled out that morphological changes in the acetabular rim may affect the various measurement methods. Irregularities of the acetabular rim termed “sonographic acetabular rim irregularity (SARI)” in ultrasound examinations of infants were described only recently, and a methodology for measuring this sonographic phenomenon was developed at the same time [6]. However, no data are yet available on the frequency of SARI as part of the DDH screening for infants in the first few months of life or regarding possible factors associated with it.

The aims of this retrospective cohort study were (1) to determine the prevalence and size of SARI during hip ultrasound examinations in infants at a specialized pediatric orthopedic outpatient clinic in the first months of life, and (2) to identify associated clinical and anamnestic factors related to this phenomenon.

## 2. Materials and Methods

### 2.1. Study Design and Study Population

The retrospective data from this cohort study were analyzed after receiving approval from the local ethics committee (Ethikkommission der Vinzenz Kliniken Wien, Gumpendorfer Straße 108, 1060 Vienna, Austria). Our study included all infants from 1 January 2014 to 31 December 2019, referred by pediatricians or orthopedic surgeons in the surrounding area of our specialized pediatric orthopedic hip clinic for further evaluation of suspected hip dysplasia or for treatment of confirmed hip dysplasia. The only patients excluded were infants with syndromic abnormalities or deformities near the hip joint, as well as cases with incomplete datasets or ultrasound images that had been incorrectly imported into our Picture Archiving and Communication System (PACS).

Out of 1103 infants in the hospital database, 3 had to be excluded due to duplicate entries resulting from a name change in the meantime. Twenty-two patients were excluded due to syndromic abnormalities. For 68 infants, the ultrasound images were not saved for evaluation, due to user errors when storing the images. Thus, a total of 1010 infants (2020 hips) were included in the study at the time of the first sonography examination. In addition to biometric data at birth, the following details were recorded: gestational age, fetal position in utero, mode of delivery, a history of DDH in first-degree relatives, and the clinical examination at the time of the initial presentation. If the DDH classification indicated that the hip joints required monitoring or treatment, these infants underwent further ultrasound examinations at intervals of 4–6 weeks (second and third hip sonography) until the completion of conservative treatment or the sonographic maturation of the hip joints during the first 4 months of life. The indication for treatment with the Pavlik harness was based strictly on the types of hip conditions requiring treatment (Type IIa minus, IIc, D, III, and IV according to Graf). Table 1 displays all of the data for the infants analyzed.

- Ultrasound Evaluation

All infant hip ultrasound examinations performed at our clinic used the standardized Graf technique [1], with the infants positioned strictly on their side in a positioning tray designed specifically for hip ultrasound, equipped with a holder for the ultrasound transducer (Sonofix^TM^ and Sonoguide™, Gebrüder Hirschbeck Inc., St. Peter am Kammersberg, Austria). The ultrasound devices used were either a Hitachi Aloka™ SSD Prosound Alpha 10 (Hitachi High-Tech Corporation, Tokyo, Japan) with a 7.5 MHz 6 cm linear transducer or a GE HealthCare LOGIQ™ P9 (GE HealthCare, Chicago, IL, USA) with a variable linear transducer (8 to 12 MHz). All data regarding the infants’ clinical history and medical records, as well as all results concerning type classifications, hip angle measurements (alpha and beta angles), and further treatment, were directly extracted from the patient records and checked for plausibility.

b. Image Evaluation

**Figure 1:**
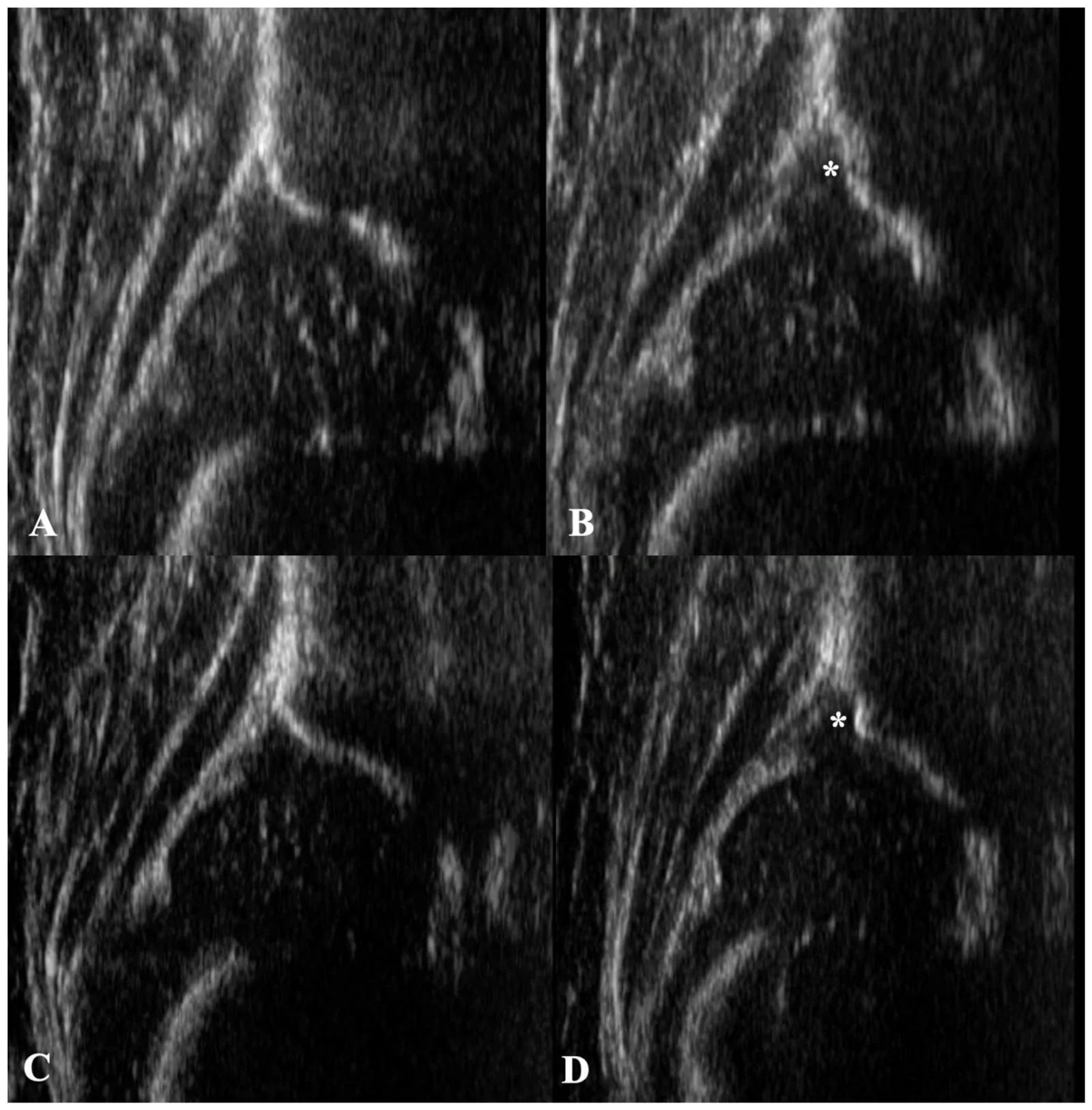
(**A**,**B**): Girl at 8 weeks of age. Type I in the right hip (**A**). Type D with SARI (*) in the left hip (**B**). (**C**,**D**): Girl at 15 weeks of age. Type I hip on the right (**C**) and Type IIb hip with SARI (*) on the left side (**D**).

All digital ultrasound examinations were reviewed together by two orthopedic specialists with a subspecialty in pediatric orthopedics to determine the presence or absence of SARI. Irregularities with concave recesses in the bony acetabular rim area, interrupting an otherwise smooth rim contour, were considered criteria for SARI. Another study has already confirmed high levels of agreement in the reporting of SARIs by different investigators (Cohen’s κ = 0.65 for inter-observer agreement and Cohen’s κ = 0.74 for intra-observer agreement) [6]. Figure 1 shows two examples of SARI in infants in our patient cohort.

**Figure 2:**
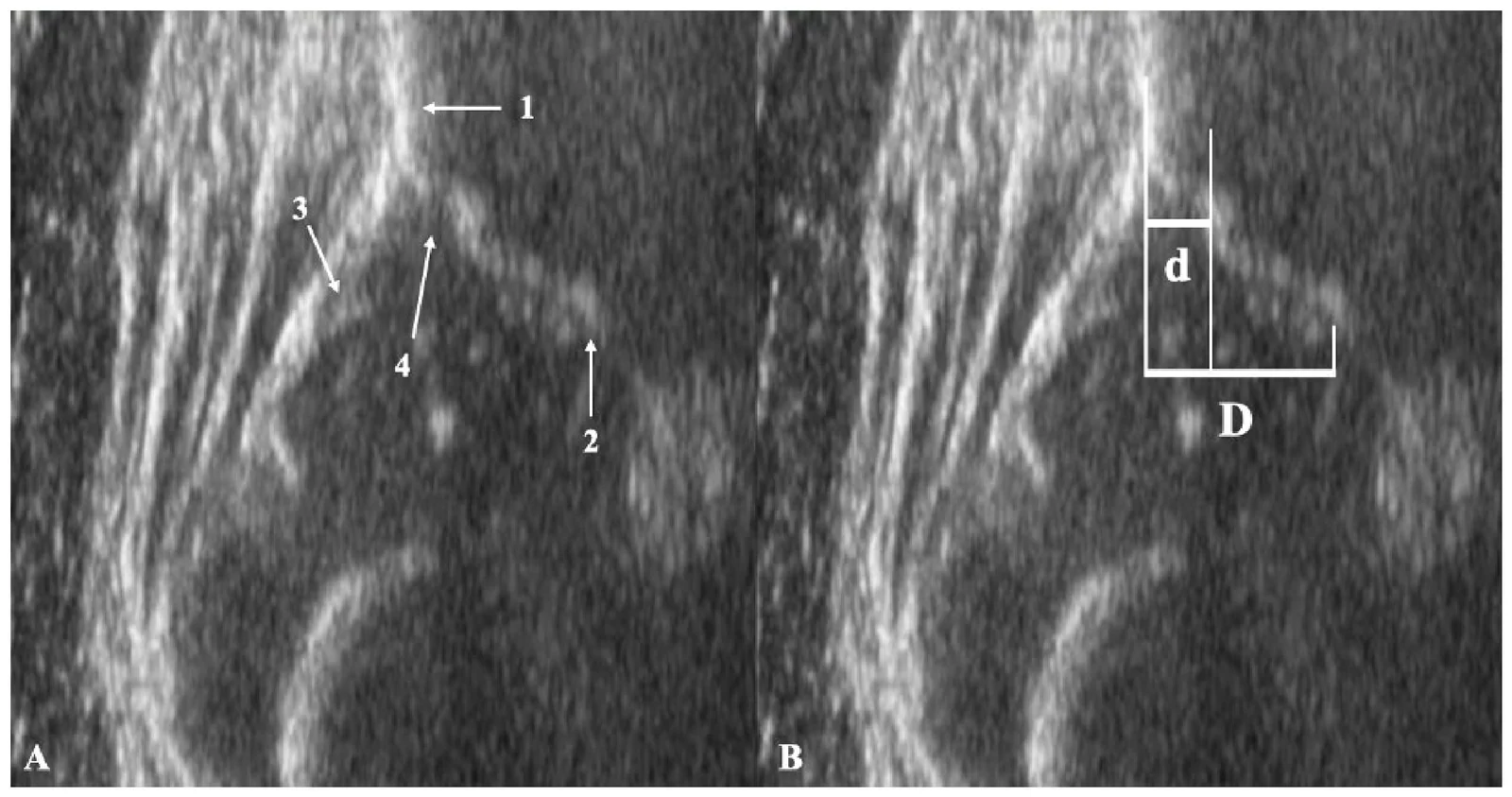
(**A**,**B**): Ultrasound image of the right hip in the standard plane, defined by a straight line passing through the iliac crests (1), the inferior border of the ilium (2), and the labrum (3). A SARI is visible (4) (**A**). To measure the size of the SARI, the width of the SARI (d) is compared with the total width of the bony coverage of the femoral head (D). SARI size = d/D (**B**).

The assessment for the presence of SARI was performed exclusively in the standard sonographic plane, as described by Graf [1]. This corresponds to the best possible frontal section through the center of the acetabulum and is defined as follows: first, by the deepest edge of the ilium in the acetabulum; second, by a vertical line through the bony iliac crest; and third, by the visualization of the fibrocartilaginous labrum in contact with the cartilaginous femoral head. SARIs were then measured based on the agreement of both examiners, using a recently published technique [6]. The relative size of the SARI was determined by measuring the distance “d” (maximum width of the concave bony recess) and the distance “D” (maximum width of the entire bony acetabular socket laterally from a tangent to the straight cut through the ilium wing, and medially to the lower edge of the ilium in the depth of the acetabulum). Figure 2 shows a SARI in the standard plane, along with the measurement of the relative SARI width.

**Figure 3:**
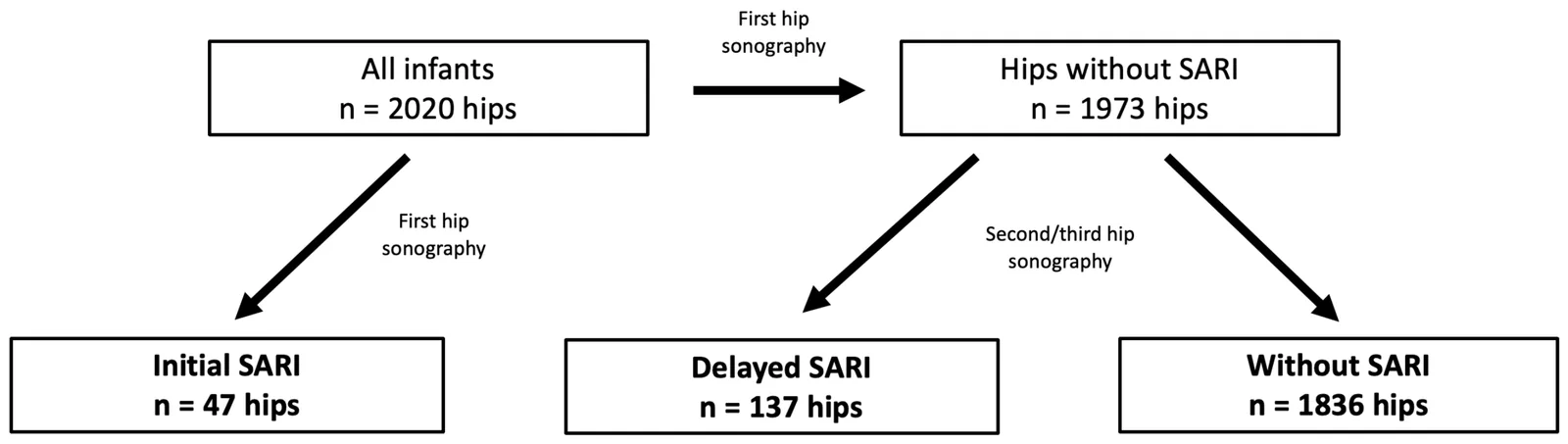
Flowchart showing the study groups.

The group of infants with SARI included, on the one hand, cases in which SARI was already evident during the first hip sonography at the hospital without prior treatment, and on the other hand, infants in whom SARI only became apparent during follow-up developmental monitoring or during Pavlik harness therapy at the second or third hip sonography. All hips with SARI accumulated over time were compared with demographic data from infants without SARI. Figure 3 shows the distribution of hip types across the study groups.

### 2.2. Statistical Analysis

Differences between the groups regarding gestational age, birth weight, and age, as well as alpha and beta angles at the time of examination, were summarized using the mean and standard deviation for normally distributed data, and with the median and 95% confidence interval for non-normally distributed data. Nominally scaled variables such as a family history of DDH, mode of delivery, sex, intrauterine presentation or side, and Graf hip classification were presented as counts and percentages. The statistical analysis for numerical data was performed using one-way ANOVA for normally distributed data and the Mann–Whitney test for non-normally distributed data; when more than 2 groups were compared and data were not normally distributed, the Kruskal–Wallis test was used to determine p-values; the chi-squared test was applied for categorical variables. A univariate and a multivariate logistic regression analysis of all recorded risk factors, comparing infants with SARI to infants without SARI, was used to predict SARI. The evaluation of the relative SARI size measurement (d/D) in comparison between initial and delayed SARI was performed using the Kruskal–Wallis test. The relative SARI ratio d/D was also correlated with the alpha and beta angle values. A significance level of 0.05 was assumed for all statistical tests. The results were analyzed using the program OriginPro^TM^ (version 2026, OriginLab Corporation, Northampton, MA, USA).

## 3. Results

Table 2 summarizes all recorded demographic, medical history, and clinical data, along with the ultrasound findings for the groups studied. At the time of the first hip sonography, 273 (13.5%) of the 2020 hip joints examined were found to have a dislocated hip type according to Graf (Type III or IV), and Pavlik harness therapy was initiated. Centered hip types (Type I, IIa, IIb, IIc, or D) without SARI were present in 1747 hips (86.5%). In 47 hip joints (2.3%) with Type I, IIa, IIc, or D, SARI was already evident at the time of the initial examination. Under Pavlik harness therapy, a centered hip type (I, IIa, IIb, IIc, or D) in combination with SARI subsequently developed in 44 primarily dislocated hips (2.18% of all 2020 hips). Additional SARIs were found in 93 primarily centered hips (4.60%) during follow-up examinations while undergoing therapy. In total, SARIs were thus observed in 184 hips (9.11% of a total of 2020 hips) and in 132 infants (13.1% of a total of 1010 infants) at the time of the initial examination or during Pavlik harness therapy in the course of the second and third sonographic follow-up examinations.

**Figure 4:**
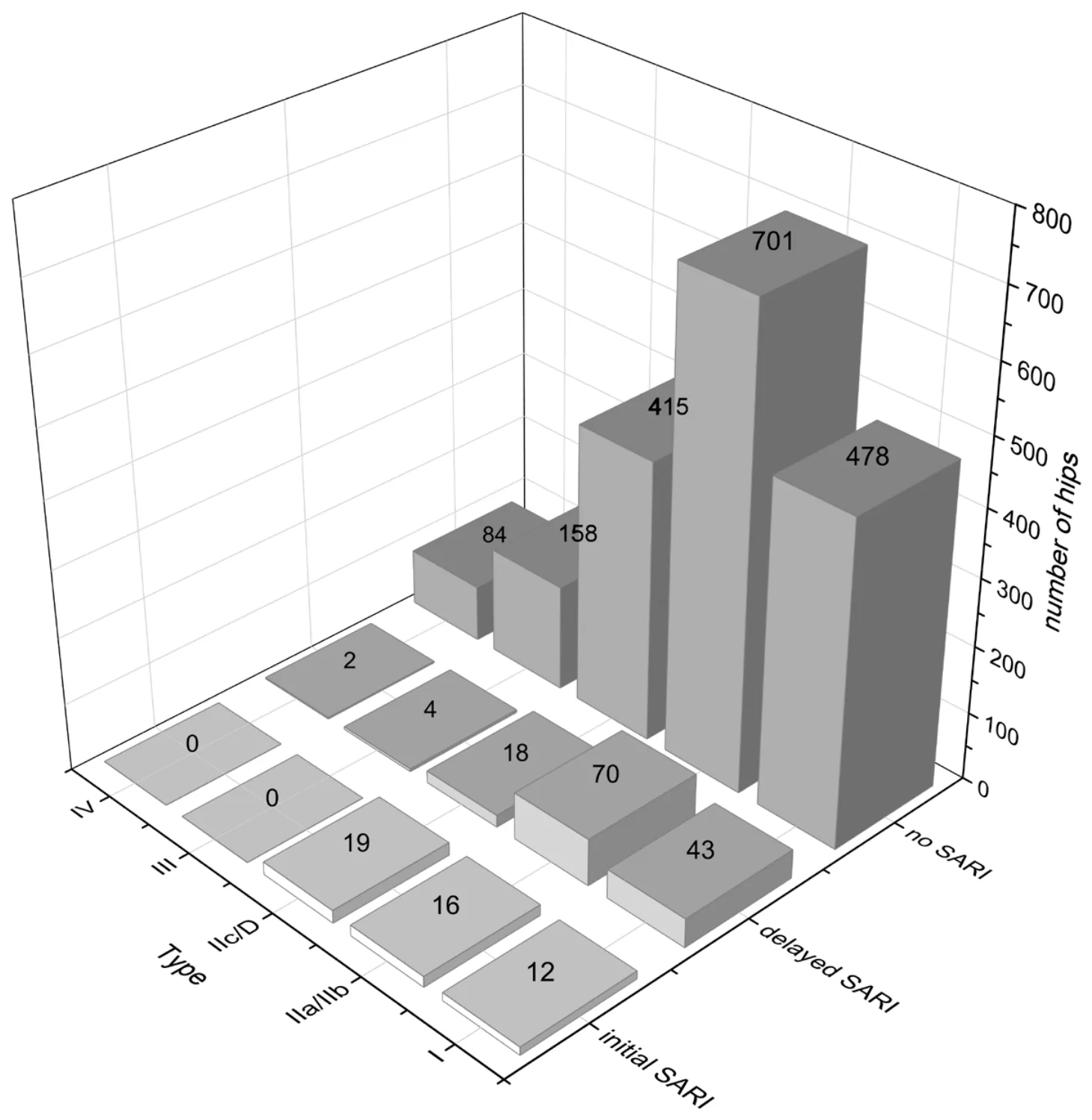
Distribution of hip types among hips with initial SARI, delayed SARI, and hips without SARI at the time of detection.

The distribution across different hip types at the time of the first manifestation of initial and delayed SARI hips was compared with hips without ultrasound findings of SARI, as shown in Figure 4.

**Figure 5:**
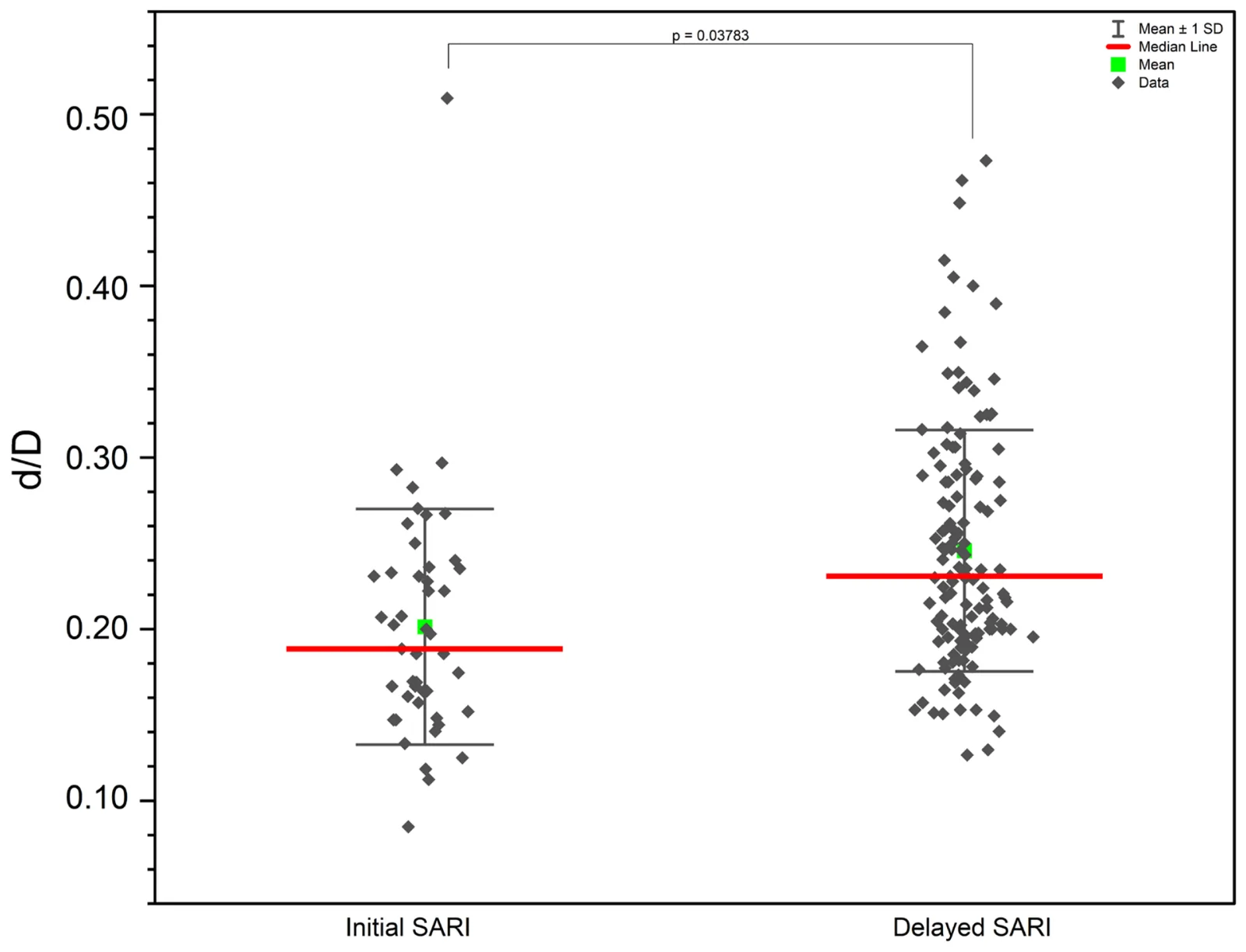
Comparison of the relative SARI size between initial SARI and delayed SARI at time of detection.

The average size of detected SARIs (either at the first hip sonography or at a later stage) was 0.23 ± 0.07 (95%CI: 0.23, 0.24). The average size of initial SARIs was 0.20 ± 0.07, and for delayed SARIs, it was 0.25 ± 0.07. Delayed SARIs under therapy were significantly larger regarding d/D compared with initial SARIs (*p* = 0.04; see Figure 5). The correlation between the SARI size d/D and the alpha or beta angle did not exceed an r^2^ of 0.01.

Univariate and multivariate logistic regressions identified cesarean section (odds ratios 1.79 and 1.88) as an independent predictor of SARI (Table 3 and Table 4). There was no synergistic effect of the variables in the multivariate logistic regression.

## 4. Discussion

Experienced sonographers specializing in infant hip examinations frequently observe atypical changes in the cartilaginous and bony roof of the acetabulum during clinical practice, but the frequency of these changes has not been described in the literature. Additionally, it remains unknown whether this sonographic phenomenon, termed SARI [6], influences the development of a mature hip joint or requires treatment beyond that indicated by the degree of dysplasia. To address these gaps, we systematically reviewed patient records from our infant hip clinic to determine the prevalence of SARI, and to identify factors associated with its occurrence.

This study demonstrates that SARI was identified in 9.1% of hips and 13.1% of all infants in a dysplasia-enriched cohort, and that this phenomenon can occur across all hip types. In decentered hip types (Types III and IV), these protrusions are visible as long as the femoral head remains in the standard plane and does not dislocate dorsally. SARI changes are most frequently observed (in more than two-thirds of cases) in hip types IIa/b, IIc, and D, which, according to Graf’s classification, are characterized by delayed maturation or a pathological degree of dysplasia. Approximately one in four SARIs in our patient population was already visible at the start of dysplasia treatment, while the majority developed during the course of treatment, particularly during the retention or late maturation phase of dysplastic hip joints. This suggests that in cases with SARI, the ossification process of the cartilaginous preformed acetabular roof may be locally delayed. Further studies are required to determine whether SARI represents merely a sonographic phenomenon or a true delay in the transition from cartilage to bone, as well as to elucidate its underlying etiology.

In our study, delayed SARIs were significantly larger than those identified at the initial hip sonography. This may be due to the fact that delayed SARIs were observed in cases treated with the Pavlik harness, where the hips were initially more dysplastic or decentered. However, we did not find a linear relationship between SARI size (d/D) and the alpha or beta angles, suggesting that SARI width does not influence the measurement of these angles according to Graf. These findings highlight the need for further investigation to clarify the clinical significance of SARI size and its relationship to hip morphology.

An interesting finding from our research is that SARIs occur approximately 1.88 times more frequently in infants born by cesarean section. Based on current evidence, it is not possible to definitively conclude that cesarean section is a truly independent risk factor for DDH and the presence of SARI. Since cesarean section frequently coexists with genuine risk factors for DDH, such as breech presentation or reduced fetal mobility, it remains practically relevant as a screening trigger, even if the causally independent mechanism has not yet been conclusively proven biologically. However, there are arguments in favor of an independent effect. Compared with vaginal delivery, elective cesarean section bypasses the labor-induced activation of fetal and neonatal glucocorticoid and catecholamine pathways [7] and prevents direct exposure to the maternal microbiota [8]. These factors play important roles in postnatal immune maturation, metabolic adaptation, and tissue maturation of various organ systems. Glucocorticoids and catecholamines are known modulators of bone and cartilage maturation; their altered peripartum dynamics following cesarean section could therefore also influence skeletal maturation processes and may lead to SARI. It is known that cortisol levels in maternal serum do not differ significantly between vaginal delivery and cesarean section, but that these levels rise significantly following vaginal delivery [9]. In addition, serum catecholamine levels are significantly lower after a cesarean section compared with vaginal births [10]. Regardless of this, there is also evidence that cesarean delivery has a cartilage-protective effect in children born in a breech presentation. A cesarean section avoids the mechanical stress on the hip joint that occurs during the active pushing phase due to the narrow birth canal [11]. However, the results of our study may suggest that a cesarean section can hormonally promote the development of SARIs, although this requires confirmation by further studies.

This retrospective study has several limitations. The patient population does not reflect a typical DDH screening cohort, as infants with dysplastic hips are referred to our specialized pediatric orthopedic outpatient clinic by maternity clinics, pediatricians, and orthopedic surgeons in the surrounding network. This referral pattern results in a higher proportion of dysplastic and dislocated hips in our cohort compared with the general population, where the incidence of DDH and the rate of DDH treatment are significantly lower [12]. Therefore, the incidence of SARI observed in this study cannot be generalized to all infants undergoing DDH screening. Nevertheless, our data indicate that SARI is a common finding among infants with hip dysplasia. The higher prevalence of dysplastic hips in our cohort allows for a more detailed investigation of factors associated with SARI. As a retrospective observational study including dysplastic and mature hip joints, infants with and without Pavlik harness therapy, and varying examination timepoints, our findings regarding the incidence of SARI should be interpreted with caution. Further research is needed to clarify how SARI develops over time, both with and without therapy, and to determine its impact on the acetabular index as measured on pelvic X-rays. For this study, based on our experience in everyday clinical practice, we chose an observation period of up to 4 months of life for newly diagnosed SARIs. Mature hip joints with bilateral Type I hips within this time period were not further monitored due to the retrospective design of the study. We therefore cannot rule out the possibility that SARI may have developed in mature hip joints without our knowledge. The quality and reliability of medical diagnostic ultrasound, particularly hip ultrasound, depend greatly on the examiner’s expertise. In this study, all infant hips were examined by experienced pediatric orthopedic specialists using a standardized ultrasound technique and consistent technical equipment to ensure reproducible results. To further enhance the reliability of SARI detection, two examiners jointly reviewed all sonograms and performed measurements as needed. We recorded only typical changes observed in the standard plane according to Graf, as these are considered to be the most significant from a biomechanical perspective. However, it is possible that irregularities in the acetabulum and its rim, located anteriorly or posteriorly to the center of the socket, may have been present in other infants but were not detected in this study.

## 5. Conclusions

SARI was identified in 9.11% of all hips and in 13.07% of all infants in a patient cohort with an increased incidence of DDH. Cesarean delivery was independently associated with SARI; further studies are needed to determine its natural history, response to treatment, and potential impact on long-term hip development.

## Figures and Tables

**Table 1:** Baseline characteristics of the patient cohort during the hip sonography examinations.

	First Hip Sonography	Second Hip Sonography	Third Hip Sonography
Infants			
Number of infants examined (n)	1010	643	502
Sex (male/female)	217/793	109/534	71/431
Age in days (mean ± SD)	27.6 ± 31.3	58.1 ± 26.3	92.4 ± 27.7
Hips			
Number of hips	2020	1286	1004
Type I (%/n)	25.0/506	44.8/576	78.8/791
Type IIa/b (%/n)	37.9/766	45.3/582	17.5/176
Type IIc/D (%/n)	23.5/475	6.2/80	1.3/13
Type III (%/n)	8.9/180	1.2/15	0.1/1
Type IV (%/n)	4.6/93	2.6/33	2.3/23
Alpha angle in degrees (mean ± SD) ^1^	54.6 ± 7.8	58.9 ± 5.7	62.9 ± 4.8
Beta angle in degrees (mean ± SD) ^1^	76.0 ± 8.0	72.4 ± 7.2	70.5 ± 7.3

^1^ Measurements are only possible for hip types in the standard plane.

**Table 2:** Demographic, anamnestic, clinical, and ultrasound examination data for all infants and hips, with and without SARI, over the observation period.

**n = 1010 Infants**	**Infants Without SARI**	**Infants with SARI**	***p*-Value**
Number of infants	878	132	
Pregnancy duration in days (mean ± SD/n)	277 ± 11/646	278 ± 8/98	0.84
Positive family history of DDH (%/n)	32.2/283	38.6/51	<0.01
Birth weight in g (mean ± SD/n)	3457 ± 523/556	3401 ± 480/86	0.37
Clinical signs suggestive of DDH (%/n)	5.9/52	8.3/11	0.29
First live birth (%/n)	27.6/242	25.8/34	0.37
Birth mode cesarean section (%/n)	10.4/91	9.1/12	0.03
Breech presentation (%/n)	14.6/128	17.4/23	0.01
Female sex (%/n)	77.7/682	84.1/111	0.09
Age in days at first sonography (mean ± SD/n)	26.8 ± 31.4/878	32.6 ± 30.3/132	<0.01
**n = 2020 hips**	**Hips without SARI**	**Hips with SARI**	
N (number of hips)	1836	184	
Left Hip (%/n)	49.6/910	54.3/100	0.62
Initial hip type			
Type I (%/n)	26.0/478	29.9/55	<0.01
Type IIa/b (%/n)	38.2/701	46.7/86	
Type IIc/D (%/n)	22.6/415	20.1/37	
Type III (%/n)	8.6/158	2.2/4	
Type IV (%/n)	4.6/84	1.1/2	
Alpha angle in degrees (mean ± SD/n) ^1^	54.8 ± 7.8/1664	56.3 ± 6.4/184	<0.01
Beta angle in degrees (mean ± SD/n ) ^1^	75.8 ± 7.9/1610	74.0 + 7.8/184	<0.01

^1^ Measurements are only possible for hip types in the standard plane.

**Table 3:** Univariate logistic regression for adjusted predictors of accumulated infants with SARI versus all infants without SARI.

Univariate Logistic Regression: SARI				
Predictors	Odds Ratio	*p*-Value	95% Confidence Interval	
			Lower	Upper
Family history of DDH (yes vs. no)	1.14	0.56	0.73	1.76
First live birth (yes vs. no)	1.18	0.53	0.70	2.00
Birth mode (cesarean section vs. vaginal delivery)	1.79	<0.01	1.16	2.75
Breech presentation (yes vs. no)	1.14	0.64	0.66	1.98
Sex (female vs. male)	1.67	0.08	0.93	2.98

**Table 4:** Multivariate logistic regression for adjusted predictors of accumulated infants with SARI versus all infants without SARI.

Multivariate Logistic Regression: SARI				
Predictors	Odds Ratio	*p*-Value	95% Confidence Interval	
			Lower	Upper
Sex (female vs. male)	1.55	0.14	0.86	2.78
Breech presentation (yes vs. no)	0.83	0.56	0.45	1.54
First live birth (yes vs. no)	1.27	0.39	0.74	2.16
Birth mode (cesarean section vs. vaginal)	1.88	<0.01	1.17	3.04
Family history of DDH (yes vs. no)	1.12	0.61	0.72	1.75

## Data Availability

The data that support the findings of this study are available from the corresponding author upon reasonable request.
